# 

**Published:** 1841-12

**Authors:** 


### CONTENTS.

PAGE.

Professor Baume's Work on First Dentition, translated by Thomas E. Bond, Jr. M. D. .......... 41

Article I.—A Treatise on Mechanical Dentistry. By Solyman Brown, M. D., D. D. S. .......... 161
Art. II.—Letter from Dr. Isaac I. Greenwood to the Baltimore Editor, 177
Art. III.—Observations on Abscess of the Antrum Maxillary. By S. P. Hullihen.......... 179
Art. IV.—Case of Ozæna, accompanied by frequent paroxysms of neuralgia faciei, cured by the extraction of a tooth. By C. A. Harris, M. D., D. D. S. .......... 184
Art. V.—Galvanism. By George G. Brewster, Esq. .......... 188
Art. VI.—Statistics of the Dental Art in Paris in 1828, .......... 192
Art. VII.—Case of Alveolar Exostosis. By S. M. Shepherd, Dentist, 193

BIBLIOGRAPHICAL NOTICES.

A Treatise on the Anatomy and Physiology of the Teeth, &c. &c. their diseases and treatment; with practical observations on artificial teeth, and rules for their construction. By David Wemyss Jobson, M. R. C. S., &c. &c. .......... 195
Every Man his own Dentist, &c. By Joseph Scott, Surgeon Dentist, 201
On the Origin and Development of the Sacs and Pulps of the Human Teeth. By John Goodsir, Jr. Surgeon, .......... 202
Three Memoirs on the Development and Structure of the Teeth and Epithelium. By Alexander Nasmyth, F. L. S., &c. &c. ..... 204
An Appeal to parliament, the medical profession, and the public, on the present state of dental surgery. By George Waite, Esq. 206
The Gums; with late discoveries on their structure, growth, connections, diseases, and sympathies. By George Waite, M. R. C. S. 206

RECENT PUBLICATIONS.

On the Structure, Economy, and Pathology of the human teeth, &c. By William Lintott, Surgeon, &c. .......... 207
The Structure, Diseases, and the Treatment of the Teeth considered; with a view to the abolition in all common cases, of the pernicious practice of tooth-drawing. By Wm. Wardrope, M. R. C. S 207
Advice on the Care of the Teeth. By Edward Saunders, Dentist, 207
Preservation of the Teeth, &c. By John Gray, Consulting Dentist, 207
Manuel Pratique d'art du dentiste, par Lefoulon, .......... 207

MISCELLANEOUS NOTICES.

Baltimore College of Dental Surgery, .......... 207
Mr. Whitewood, .......... 208
Dental works in progress, .......... 208
Anomalous Dentition, .......... 208
Dr. E. B. Gardette and the American Society of Dental Surgeons, 209
Plugging Teeth, .......... 210
Animal Magnetism, .......... 212

